# Sodium, phosphate, and nitrite: from brine to brain, what matters?

**DOI:** 10.1093/af/vfag002

**Published:** 2026-01-25

**Authors:** Henk W Hoogenkamp, Henk R Hoogenkamp

**Affiliations:** Independent Researchers; Independent Researchers

**Keywords:** nitrite, phosphate, potassium, salt reduction, sodium chloride

Implications

- In savory products, a practical sequence is to build umami first, using glutamate where it fits technically and sensorially, then, add partial potassium chloride and process or sensory tweaks to hit the sodium target.
- It is more practical to set sodium reduction goals by category and with more attention for high intake groups. These strategies best go hand-in-hand with increased dietary potassium intake through vegetables, pulses, fruits, and nuts.
- For curing, risk should be managed by exposure, matrix, and process, maintain color, flavor, and botulism control, suppress nitrosamines with ascorbate, careful time and temperature, and residual limits, and offer transparency in vegetable derived nitrite systems.
- Phosphate additives have high bioavailability, and therefore, category limits and balances should be set which enables clear targets for functional alternatives like hydrocolloids, fibers, and enzymes, minimizing compromise on safety, quality, or affordability.

## Introduction

Consumers report wanting less sodium yet reject products that fall short on taste. Public health agencies have responded by tightening intake guidance to curb diet-related disease. For food product developers, salt’s multifunctionality complicates meaningful reduction. A more pragmatic approach is to tune perception first, then, engineer the safety and texture that salt used to provide.

## The Sodium Problem: Burden, Drivers, and What’s Working Now

Hypertension affects about 1.28 billion adults aged 30 to 79 worldwide, with the majority living in low and middle income countries, which highlights the scale of the exposure to excess dietary sodium in real world settings (World Health Organization, 2023a).

In high income countries the main source of sodium does not come from the saltshaker but from processed and prepared foods. In the United States, people average more than 3.3 g sodium per day, while the guideline is below 2.3 g (5.8 g of table salt). For reference, 1 g sodium is about 2.5 g sodium chloride. (Center for Disease Control, 2024) Exposure is not even, and intake seems to be socially patterned. A systematic review across 19 high income countries found that adults with lower socioeconomic status consumed about 14% more sodium than higher status groups, which contributes to unequal burdens of diet related disease (Mestral et al., 2017).

Marketing and availability shape sodium intake, particularly in youth. The World Health Organization (WHO, 2012) recommends mandatory restrictions on the marketing of foods and beverages high in saturated fat, trans fat, free sugars, and salt to reduce harmful exposure and intake among children and adolescents (World Health Organization, 2023b). Evidence cited by the guideline show that reducing exposure to unhealthy food marketing leads to improvements in food preferences and choices in young people (Harris and Graff, 2011).

## Salt and Phosphate Reduction, Why It Is Simple to Say, and Complicated to Do

In most meat and savory systems, salt quietly does a lot of heavy lifting: it drives overall taste, suppresses bitterness, enhances protein extraction and gelation, binds water, and helps with microbial control, all at low cost. Lowering sodium chloride can reduce overall flavor, unmask bitter or chemical notes, weaken protein extraction and water holding, and in general shorten shelf life across bakery, sauces, brines, marinades, and processed meat or plant based analogues (Kilcast and den Ridder, 2007).

On the ingredient side, potassium chloride is the familiar partial replacer, yet at useful levels it often leaves bitter or metallic notes, which limits its use as a stand-alone replacer in many categories. Monosodium glutamate (MSG) provides umami with far less sodium per gram than salt, about 12% sodium vs. about 39% in sodium chloride, and it increases savory perception through glutamate binding that can substitute for part of the salt signal in many matrices. In soups, sauces, meats, and snacks, replacing a portion of sodium chloride with MSG can deliver meaningful sodium cuts while preserving liking and saltiness. Other tools, beyond potassium and MSG have been developed, and are discussed later on in this article, but in practice all these tools work best in blends that respect the product’s sensory expectations.

When sodium chloride steps down as a microbial barrier, other hurdles must be erected. In cooked and ready-to-eat meats, lactate or acetate salts (often potassium based to avoid adding sodium) suppress pathogens and spoilage organisms. Bacteriocins such as nisin or pediocin provide targeted inhibition against Gram-positive organisms including *Listeria monocytogenes* (­Cotter et al., 2005). Water activity control remains foundational and can be tuned with non-sodium salts, for example potassium lactate or acetate, glycerol, or selected polyols, to maintain stability at lower sodium. Processing and structure tools can also carry part of the safety load, high pressure processing, ultrasound and pulsed electric fields improve lethality or accelerate uniform uptake of curing agents in reduced salt matrices but cannot be used on all product types. If phosphates are reduced alongside sodium for clean label or regulatory reasons, water holding and sliceability can be recovered with hydrocolloids and fibers, i.e., citrus fiber, alginates, carrageenan, or pre-gelled starches. Antioxidant systems, for example ascorbate, tocopherols, and polyphenol extracts, should be re-evaluated as sodium levels and water activity shift, because changes in ionic strength, competing ions, or replacement ingredients can alter lipid and pigment oxidation behavior (Inguglia et al., 2023).

## Clinical Perspective: Blood Pressure, Potassium Balance, and Health Endpoints

Elevated blood pressure in (young) adulthood is common and clinically relevant. In the CARDIA study (Coronary Artery Risk Development in Young Adults), young adults with elevated blood pressure or stage 1 or stage 2 hypertension before age 40 had higher subsequent risk of cardiovascular events than those with normal blood pressure, which supports earlier dietary and lifestyle interventions that include sodium reduction (Yano et al., 2018).

Lowering sodium reduces blood pressure within weeks in both hypertensive and normotensive adults, with larger effects in salt sensitive and older populations, which translates into meaningful reductions in cardiovascular events at scale when population sodium intake falls (He et al., 2013).

Dietary potassium counterbalances sodium’s effects. A WHO commissioned meta-analysis found that higher potassium intake lowers blood pressure and is associated with lower stroke risk in adults. Most populations under-consume potassium, so national guidance treats it as a nutrient of public health concern. Whole food sources, vegetables, pulses, fruits, and nuts, are preferred (Aburto et al., 2013).

In people with chronic kidney disease, salt reduction lowers blood pressure and albuminuria in the short term, which are markers for slower progression. Meta analyses suggest general benefits for renal-related outcomes, although long term trials are still needed for hard endpoints (Shi et al., 2022).

Gastric cancer risk is also linked to high salt exposures (World Cancer Research Fund, 2018). The World Cancer Research Fund concluded that foods preserved by salting, for example salted fish and some pickled vegetables, are a cause of stomach cancer. Epidemiologic work indicates that high salt intake can damage gastric mucosa, can enhance colonization or virulence of *Helicobacter pylori*, and can act synergistically with nitrosating conditions, which together raise risk (Balendra et al., 2023).

On the flip side, sodium is essential for normal physiology, which argues for pragmatic targets rather than uniform very low intakes for everyone. Meta-analysis shows that aggressive sodium restriction can activate counter regulatory hormonal systems and raise circulating lipids in the short term. Therefore, population guidance should focus on bringing high intakes down while allowing individual clinical care, particularly for people with complications or who are on complex diets (Farquhar et al., 2015).

## Current Responses Span Policy, Communication, and Formulation

On the policy side, structural levers include category targets, clearer labeling, restrictions on marketing to children, and incentives that focus on the highest intake categories and groups. Many national frameworks use incremental, category-based reduction strategies rather than a single universal number. Typical interim targets aim for 10–20% sodium reduction within major contributing categories over 3–5 years, with long-term population goals aligning with mean intakes below 2.3 g sodium/day in adults and lower for high-risk groups.

More specifically, the United States Food and Drug Administration (FDA) set voluntary, measurable targets across more than one hundred categories. Moreover, the progressive United Kingdom program provides precedent for stepwise reformulation with measurable falls in population salt intake (He et al., 2013; Food and Drug Administration, 2021).

From the consumer communication perspective, education is an uphill battle but remains paramount. Prioritizing high-intake populations and pairing sodium reduction with higher dietary potassium from fruits and vegetables is prudent; potassium blunts sodium’s physiological effects and is linked to lower cardiovascular mortality (Aburto et al., 2013). On the flip-side, consumer communication invites misuse and will eventually backfire: e.g., consumers often view “sea salt” more positively than “salt,” even though both deliver sodium, reinforcing salt preference instead of reducing it (Watson, 2011).

On the technology side, three strategies are delivering reductions while protecting acceptance; First, gradual reduction with small and sustained step downs that retrain consumer expectations. Second, chloride swapping with partial replacement by potassium salts, magnesium salts. Lastly, umami/flavor design to maintain perceived salt intensity at lower sodium. There are also some complementary levers that can help make less feel like more; smaller or hollow salt particles to boost early dissolution and perceived saltiness (Jiang et al., 2025), aroma–taste congruency, umami cues such as soy, Parmesan, and green tea, to elevate salt perception via cross modal integration (Thomas-Danguin et al., 2019). The low-hanging and affordable fruit remains co-crystallized NaCl and KCl which retains familiar user-friendliness and functionality with limited bitterness (Lorén et al., 2023). In many processed foods and meats, a 70:30 NaCl to KCl ratio is a common starting point that is then tuned to sensory tolerance and product suitability (Nurmilah et al., 2022).

## Sensory and Taste-Science Levers

In practice, sodium reduction efforts usually run into sensory and cost limits rather than a lack of technical options. The tongue and brain judge complete flavor through interacting cues that formulators can design. Due to advances in analytical technologies and understanding of complex flavor interactions; some old fermentation-based flavoring technologies are now better understood and subsequently allows for ingredient companies to precisely develop the next generation of ingredients. With larger trained sensory panels combined with instrumented flavor data, machine-learning models can be trained to pick up complex cross-modal predictors of saltiness. Pinpointed aroma congruency, temporal dominance shifts, or mouthfeel changes can flag promising low-sodium directions before bench testing, especially in matrices where interactions are too complex or time-consuming for humans to explore manually. That being said the technologies being pushed as innovations (some listed below) are mainly new sources of more defined fractions of ingredients and not entirely new categories on their own.

- **Enhancing salt perception whilst reducing net sodium**. Pair glutamate with 5′-nucleotides (IMP, GMP—I+G), to amplify saltiness (Amado et al., 2024). Use aroma–taste congruency, for example, smoked, grilled and fried notes in savory foods to raise perceived salt intensity through cross-modal integration.
- **Kokumi, umami, and mouthfulness**. Kokumi compounds such as γ-glutamyl peptides, glutathione and other di-/tri-peptides act through the calcium-sensing receptor to increase perception of thickness, continuity, and lingering savor (Miyaki et al., 2015). Used alongside glutamate and 5′-nucleotides (IMP, GMP), di-/tri-peptides can make reduced-sodium foods taste full rather than thin. Fermented soy pastes, aged cheeses, and yeast fractions naturally supply these molecules but it is industrially feasible to specifically filter out these peptide fractions from the complex sources using a combination of ultra- and nano-filtration.
- **Delivering the same signal with less sodium**. Engineer salt particles to dissolve faster by using small, porous, or hollow microspheres, and consider co-crystallized NaCl/KCl so first contact delivers a strong pulse while total sodium falls (Lorén et al., 2023). This allows for inhomogeneous distribution, with more salt near surfaces to preserve the initial saltiness perception with less in the bulk.
- **Managing bitterness when swapping chloride sources**. Potassium salts can leave a bitter or a metallic tail. Yeast-derived fractions, mushroom extracts, glycyrrhizin derivatives, and targeted bitter blockers can mask these notes while keeping labels acceptable (Lu et al., 2022).
- **Plant and clean-label levers**. Seaweed and mushroom extracts bring naturally present salts, short peptides, glutamates and 5′-nucleotides (Phat et al., 2016). Koji-style ferments provide umami scaffolds with minimal sodium contribution, especially useful in plant-based meats and cheeses.

## Curing Chemistry, Nitrite, and Safety Innovation

Public and regulatory pressure to curtail nitrite, and in some cases phosphate, seems to have grown out of general meat-demonization, ultra-risk averse framing and naturalness heuristics. The constructive response is a technical one; use quantitative, process specific risk assessment and newer tools so that nitrite can be reduced or replaced without compromising safety, color, and flavor. Nitrite is central to the antimicrobial hurdle against *Clostridium botulinum* but also provides cured color and flavor. Yet, under specific processing and cooking conditions nitrite can form nitrosamines which is at the center of the epidemiologic association between processed meat and colorectal cancer have driven tighter oversight and innovation (Honikel, 2008; Bouvard et al., 2015).

Curing is feels familiar to both the public and processors but is increasingly contested, which makes dietary context and label language important. Plant foods supply most dietary nitrate, which is reduced by the oral microbiome to nitrite and further to nitric oxide, a molecule that supports vascular function in certain contexts (Hord et al., 2009). Against this physiology sits a labeling crux. Products labeled “uncured” or “no-nitrite-added” often use vegetable nitrate with starter cultures to generate nitrite *in situ*, which means the fundamental curing chemistry in the meat matrix is identical to conventional curing. U.S. regulators have acknowledged the potential for consumer confusion and have considered changes to the uncured and no-nitrite-added statements for products cured with vegetable sources, while maintaining that risk is governed by dose, matrix, time and temperature history, pH, and residuals rather than the origin of nitrite (Food Safety Inspection Services, 2020). Clearer, not cleaner, statements regarding vegetable derived curing with culture mediated nitrite generation, would better align labels with mechanisms and reduce confusion (Bedale et al., 2016). Yet years of brands jostling for shelf-space using front-of-pack claims have shaped nitrite perception more than risk, and it is not evident to the authors how to unwind this.

Regardless of labeling strategy, a technical mitigation toolkit within conventional curing is readily available and widely applied. Where nitrite is used, nitrosamine formation can be, and is already, minimized with layered controls (Honikel, 2008). These include ascorbate or erythorbate to accelerate nitric oxide formation and quench nitrosating species, control ingoing dose and residual nitrite with validated end of process targets and reject limits. Next to this, it has been common practice to manage pH and water activity to maintain microbial hurdles without favoring nitrosation. Support from antioxidants such as tocopherols and selected phenolic sources, or refined smoke condensates with standardized composition may also offer another level of control. It is worth noting, that smoke condensates have recently come under regulatory scrutiny in Europe due to genotoxicity concerns. Readers are referred to the European Food Safety Authority’s 2023 review (Younes et al., 2023). Adopting gentler cook regimes that avoid high temperature frying and direct flame where possible will help avoid nitrosamine formation but is nearly impossible to implement as this means touching on consumer preparatory skills. However, even this robust level of control alone is not sufficient to rebuild consumer trust or fight misinformation.

**Sidebar**In the U.S., pumped bacon must use 120 ppm ingoing nitrite and 550 ppm ascorbate/erythorbate, an inhibitor combo adopted specifically to suppress nitrosamine formation during frying; for decades, FSIS also sampled fried bacon to verify low nitrosamines, and levels fell after these controls were adopted. Dry-cured bacon, by contrast, may use higher ingoing nitrite and is not required to include ascorbate, and retail-exempt shops are not under continuous federal inspection nor required to run HACCP systems like official establishments (Code of Federal Regulations, 2025). Morevoer, pan-frying/over-browning raises nitrosamines while microwave cooking lowers them, and the picture is clear: consistency and mitigation come from validated controls, not from nostalgia.

There are alternative nitrate generation routes being developed and standardized. For example, culture-based systems pair nitrate reducing starters, for example *Staphylococcus* species and selected lactic acid bacteria, with standardized vegetable nitrate to generate nitrite and nitric oxide *in situ*, which can deliver cured color and flavor with improved label optics but does not change the compositional chemistry. Pre-cured plant extracts and controlled fermentates can supply cured like sensory attributes and antioxidant support at reduced nitrite levels, and amino acid routes that engage endogenous nitric oxide pathways remain promising but early for commercial use (Bedale et al., 2016; ­Siekmann et al.,2021).

## Phosphate Additives: Functionality, Bioavailability, and Health Context

In both emulsified and whole-muscle products, phosphate salts are still among the main tools for water binding, pH control, ionic strength, emulsion stability, and freeze–thaw tolerance. They increase extraction of the myofibrillar protein into the aqueous phase, stabilize fat/water matrices, reduce cooking loss, and support sliceability. These contributions are difficult to replace without compromising purge, yield, and microbial robustness in injected, chopped, and restructured systems.

Unlike organic phosphates bound in whole foods, added inorganic phosphate salts have very high bioavailability (typically >80–90%) (Karp et al., 2012). In people with impaired renal function, higher circulating phosphate and fibroblast growth factor 23 are associated with vascular calcification and overall increased cardiovascular risk. Phosphate additive prevalence and potential exposure implications are examined in a recent comprehensive assessment, which concludes that phosphate additives are widespread across U.S. packaged foods and that their contribution to total phosphorus intake is substantial enough to warrant clearer labelling of phosphorus content (Dunford and Calvo, 2025).

Reformulation pressure is heightened by global inconsistency in maximum allowable addition levels, ongoing “clean label” policies, and regulatory uncertainty surrounding “ultra-processed foods.” Legal limits differ widely across global markets, which complicates transparent interpretation of both safety and functionality. Practical industry responses, listed in the sidebar, currently go against the pressure brought by “clean-label” and general cost reduction. In any case, any reduction must be validated for water activity, purge, oxidation stability, and pathogen control, so as not to trade hypothetical long-term risks for acute food safety or waste problems.

**Sidebar**Functional alternatives when reducing phosphates:

- Hydrocolloids & fibers: Carrageenan, alginate, pectin, citrus fiber; improve water binding and viscosity, but can alter bite and opacity.
- Starch systems: Pre-gelled or modified starches to support yield; risk of pastiness if overused.
- Enzymatic tools: Transglutaminase for network strengthening in whole-muscle or restructured systems.
- Process tools: High-pressure tumbling, longer equilibration time, improved injection uniformity.
- Limitations: None replicate phosphate’s combined pH + ionic strength + chelation functions; all require validation for yields, safety, and oxidation.

## Worth Its Salt: Closing Reflections on Taste, Risk, and Rent

Sodium reduction is necessary but is far from simple. In the end, taste, safety, and texture set hard boundaries that rhetoric cannot move. The most reliable path remains non-revolutionary and practical: gradual step-downs that retrain expectations, better overall diet quality with higher potassium, and flavor design that preserves acceptance. In processed meats, the nitrite question demands not only nuance rather than marketing slogans but also modernization of curing while protecting microbial safety and color/flavor integrity. Like nitrite, phosphates merit balanced science-based limits that reflect their high bioavailability and undeniable functionality. It is imperative to keep the marketing teams out of the door in the early-stages when attempting to align consumer expectation, technical feasibility, and risk prioritization or risk chasing zero-sum purity narratives with front of pack claims.

Sadly, reformulation does not occur in a vacuum; it unfolds in a marketplace. Salt is cheap and most of its substitutes are usually not. That price gap what one might refer to as “flavor inflation”: high-tech enhancers coupled to immaculate narratives, flavors hidden under the processing aid veil and opaque pricing. This can be a challenge for premium markets, let al.ne the markets serving the highest risk low income demography. When flavor companies get involved, margin-stacking becomes the model, reformulation slows due to lower transparency, access narrows, and the public-health upside evaporates.

If we care about health and honesty, we should pull the most efficient levers first. Effective and affordable umami tools such as monosodium glutamate should be considered early in reformulation, rather than bypassed in favor of more expensive I + G systems marketed as “MSG-free.” Such tick-box practices inflate formulation costs without improving risk profiles. What really matters is aligning technical solutions, risk prioritization, and pricing rather than front-of-pack narratives.

## Author Biographies

**Henk W. Hoogenkamp**, Author and industry advisor specializing in protein functionality, meat science, and cost focused reformulation. He helps companies lower their formulation costs while protecting texture, flavor in meat and plant-based products. His career spans leadership and advisory roles with global food and ingredient firms, including President of Friesland Campina USA and Senior Director of Strategic Technology at DuPont Protein BioScience, and has authored multiple articles and books on ­protein technologies.

**Henk R. Hoogenkamp**, Biochemist and founder of Future Foods, a technology scouting firm that develops ingredient and technology innovation programs for companies worldwide. Co-founder and former chief product officer at Moolec Science, working at the interface of molecular farming and scalable animal protein production in plants. Education includes a PhD in animal-derived biomaterials for regenerative medicine from Radboud University Nijmegen, a Master of Applied Science in life sciences, and a Bachelor of Applied Science in biochemistry.
